# Using Personas in the development of eHealth interventions for chronic pain: A scoping review and narrative synthesis

**DOI:** 10.1016/j.invent.2023.100619

**Published:** 2023-04-03

**Authors:** Sara Laureen Bartels, Afra S. Taygar, Sophie I. Johnsson, Suzanne Petersson, Ida Flink, Katja Boersma, Lance M. McCracken, Rikard K. Wicksell

**Affiliations:** a Department of Clinical Neuroscience, Karolinska Institutet, Stockholm, Sweden; b Institute of Social Sciences, Uskudar University, Istanbul, Turkey; c Department of Medicine and Optometry, Linnaeus University, Kalmar, Region Kalmar County, Sweden; d Center for Health and Medical Psychology (CHAMP), School of Law, Psychology, and Social Work, Örebro University, Sweden; e Division of Clinical Psychology, Department of Psychology, Uppsala University, Uppsala, Sweden; f Pain clinic, Capio St. Göran Hospital, Stockholm, Sweden

**Keywords:** User research, Personas, Internet, Intervention, Development, Chronic pain

## Abstract

**Objectives:**

Behavioral eHealth interventions can enhance self-management and improve well-being in people with chronic pain. The development of these interventions calls for a user-centered approach to ensure that patient needs are appreciated. However, it may be challenging to involve patients; particularly during the early stages of the process. Fictional user profiles, known as *Personas*, can represent needs and guide designing eHealth interventions. This article provides a comprehensive overview of the use of Personas in the development of behavioral eHealth interventions for people with chronic pain with the aim to identify benefits and challenges.

**Methods:**

Bibliographic databases (Medline, Web of Science Core Collection, PsycInfo, CINAHL) and registries (PubMed Central, medaRxiv) were systematically searched. In a double-reviewing process, *n* = 6830 hits and *n* = 351 full-texts were screened and read. Ten peer-reviewed studies published between 2017 and 2022 were included in the narrative synthesis.

**Findings:**

Ten studies reported using “Pain Personas” in the development of eHealth interventions for such purposes as to gain a shared understanding of the user and to discuss solutions in team meetings, or for patients to identify with (if Personas are included in the intervention). Personas were based on qualitative and/or quantitative data. However, the procedure for creating Personas was only described in half of the included studies (*n* = 5). These five studies provided descriptive details of the Personas (i.e., picture, name, narrative of their pain behavior, technological skills, and motivation).

**Conclusions:**

Although Personas have been used by pain researchers in recent projects and were highlighted as an important ingredient in the development process, available design guidelines for the creation and use of Personas are not followed or communicated transparently. Benefits and challenges when using Personas in the development of eHealth interventions for people with chronic pain are discussed to support future eHealth efforts and to improve the quality of eHealth innovation in the field of pain.

## Highlights

• Personas are increasingly used by researchers for scientific innovation.
• More scientific rigor and transparency on the Personas process is needed.
• Pain Personas reflect users’ background, behaviors, thoughts, and technology use.
• Guidelines for the use of Pain Personas are needed and should be evaluated.

## 1 Introduction

In healthcare and health science, there is an urgent call for personalized, tailored interventions combined with a biopsychosocial care approach (Evers et al., 2014; Ricciardi and Boccia, 2017; Wade and Halligan, 2017). Digital solutions and innovations, also referred to as eHealth, are uniquely suited to answer this call (Car et al., 2017). The World Health Organization (WHO) defines eHealth as ‘cost-effective and secure use of information and communication technologies in support of health and health-related fields, including health-care services, health surveillance, health literature, and health education, knowledge, and research’ (WHO, 2022). Benefits of eHealth include improved access and quality of care, and more time- and cost-efficient care (Granja et al., 2018; LeBlanc et al., 2020). eHealth has therefore the potential to impact public health on a large scale (Grady et al., 2018).

When developing eHealth interventions, user input and patient and public involvement (PPI) is essential (Skivington et al., 2021). The framework from the UK Medical Research Council and National Institute for Health Research emphasizes that meaningful engagement of patients as key stakeholders can maximize the potential of novel interventions that are likely to positively impact health (Skivington et al., 2021). However, a number of financial, organizational, and sociopolitical barriers can limit PPI (Boivin et al., 2010), particularly in the early stages of a research project. For instance, professionals might have negative attitudes about patient contributions and academics may have doubts about the benefits of user input (Ocloo et al., 2021). Furthermore, researchers might experience uncertainty about how to conduct PPI, in terms of representativeness and diversity of the target population, high workload or time constraints of clinical staff, patient consent, or ethical issues (Ocloo et al., 2021). Also, it may be challenging to involve users before obtaining ethical approval, at a stage where interventions including eHealth prototypes are usually being prepared. Nevertheless, it is important to keep users and their needs in mind during the early stage of development, and continuously throughout evaluation and implementation phases.

**Fig. 1:**
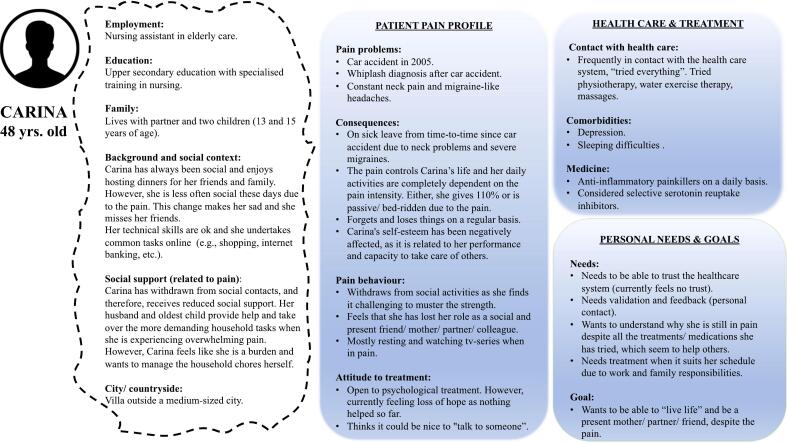
Persona with chronic pain (used but not previously published as part of the DAHLIA project, Bartels et al., 2022).

Fictional and exemplary user profiles, scenarios, or *Personas*, serve as a way of representing prospective users. In the current review, the focus lies on Personas, defined as fictitious, specific, concrete representations of target users (Pruitt and Adlin, 2010). A Persona is, thus, a description of a fictious person representing a defined user group, detailing their characteristics (e.g., name, photo, symptoms, psychological reactions, preferences), and context (see Fig. 1: Example for a Persona with chronic pain).

For several decades, Personas have been increasingly used in research and product design (Goh et al., 2017), representing a memorable, engaging, and actionable image serving as a design target (Pruitt and Adlin, 2010). As described in Goh et al. (2017), the contemporary term ‘Persona’ was first introduced in the late 90s (Cooper, 1999), referring to a ‘creative way of constructing a type of person who would use a particular computer application’ (p. 606) (Goh et al., 2017). It should be noted that, while Personas are fictional in terms of their detailed narrative, the methodology itself builds on existing data sources from the literature or clinical observations. Nowadays, in organizations and companies developing commercial products and services, Personas represent a common method to incorporate a focus on users. Practical guides for user experience (UX) designers discussing the essential Persona Lifecycle are available and provide a framework for creating Personas in five steps (i.e., family planning, conception and gestation, birth and maturation, adulthood, lifetime achievement and retirement) (Adlin and Pruitt, 2010).

In several research fields, Personas are used in the development of eHealth interventions. For instance, two Personas illustrating cancer survivors were integrated in the design of a stress management app to ensure that user voices were taken into account (Børøsund et al., 2018). In this project, Personas were created based on insights gained through interviews and included cancer survivors' background and challenges, their use of technology, as well as needs and requirements for an electronic stress management intervention. However, authors did not elaborate on exactly how Personas were used in the design and development phase (Børøsund et al., 2018). In another study, digital diary data from people with self-reported obesity was used to create five Personas to support the prospective development of an intervention for binge eating and weight management (Graham et al., 2021). Similarly, Personas were an integrated part of designing eHealth solutions in various academic settings, including cancer (Benedict et al., 2021; Nygren et al., 2017; Papadakos et al., 2017; Wärnestål et al., 2017), patients with complex needs (Bhattacharyya et al., 2019), chronic illness care (Fore et al., 2013), multiple sclerosis (Giunti et al., 2018), hypertension self-management (Duan et al., 2020), cardiovascular disease medication intake (Haldane et al., 2019), heart failure (Holden et al., 2017), autism spectrum disorder (Silva and Teixeira, 2019), sexual health behavior (Strong et al., 2020; Wray et al., 2019), and eHealth skills of healthcare professionals (Bierbooms et al., 2021). In these studies, methodologies to create Personas varied, relying, for instance, on a literature review (Benedict et al., 2021), ethnographic observations (Papadakos et al., 2017), hierarchical cluster analysis (Holden et al., 2017), qualitative and quantitative methods (Duan et al., 2020), or patients developing Personas in focus groups (Wray et al., 2019). These examples show that Personas are commonly used as a tool by the development team in brainstorm meetings and the intervention design decision-making process (Giunti et al., 2018; Haldane et al., 2019; Nygren et al., 2017; Silva and Teixeira, 2019; Wray et al., 2019).

In addition to using Personas as a user representation for the development team, Personas can also be helpful in translating complex information into actionable steps (Brooks and Greer, 2014). Thus, patients might benefit from the use of Personas *within* digital interventions; Personas illustrating reactions that are familiar to the patient can be validating, and may also result in a better understanding of the treatment rationale. For instance, a Person can be introduced to patients through a short video, quote, or descriptive text demonstrating how the Persona learned and implement behavioral exercises into their day-to-day life. As part of tailored interventions, Personas have been shown to enhance patient engagement and satisfaction (Serio et al., 2015). Taken together, Personas hold promise in both treatment development and delivery.

The pain field represents an area where the use of Personas could make a significant contribution to intervention development, as there appear to be a virtual explosion of eHealth apps and web-based programs in this area (Lau et al., 2020; Moman et al., 2019). Chronic pain is recognized as a major public health problem, affecting about one third of the adult population, and incurring huge personal and societal costs (Dueñas et al., 2016). eHealth interventions are seen as a way to improve access to evidence-based care in pain populations (Slattery et al., 2019), especially behavioral or psychosocial interventions based on cognitive behavioral models to enhance self-management, functioning, and well-being in people with chronic pain (McCracken and Morley, 2014).

To promote the use of the Personas methodology in a systematic way and enhance the quality of the eHealth intervention development process, it is important to know if and how Personas are currently being used in research on behavioral eHealth approaches for chronic pain. To date, it is unclear what rationale pain researchers have for using Personas, what the process of Personas creation looks like, what characteristics Personas with chronic pain have, and how Personas are utilized in the eHealth development process. To our knowledge, no peer-reviewed, published review on the use of Personas in the context of digital intervention for chronic pain field exists.

Hence, the aim of this study is to provide a comprehensive overview of the use of Personas in the development of behavioral eHealth interventions for people with chronic pain by systematically reviewing and narratively synthesizing current Persona efforts. With a focus on the development of eHealth intervention for chronic pain, the following specific research questions will be answered: i) Why were Personas used? (i.e., rationale) ii) How were Personas created? (i.e., methodology used to establish Personas) iii) What do Pain Personas look like? (i.e., characteristics and details), and 4) How were Personas used? (i.e., process and steps of use). Potential benefits and challenges when using this methodology, as well as considerations for how to characterize Personas of individuals with chronic pain, are discussed to inform future developments of eHealth interventions and promote a systematic and rigorous use of the Personas methodology in the field of chronic pain.

## 2 Methods

### 2.1 Search strategy

The bibliographic databases Medline, Web of Science Core Collection, PsycInfo, and CINAHL, and registers PubMed Central and medaRxiv were systematically searched by the Search Group, a service offered for researchers working at Karolinska Institutet, to identify studies on digital behavioral interventions for chronic pain. Databases were selected to cover literature from both broad (e.g., Medline, Web of Science) and specific research areas (e.g., PsycInfo, Cinahl). Registries were used to be able to search in the full-text for the Personas-concept.

The following search terms were used: ‘internet or digital’, AND ‘intervention’, AND ‘chronic/ persistent/ long-term pain’. ‘Personas’ was not included in the search strategy as it was unlikely to be mentioned in the title or abstract, thus would limit the search. Appendix A presents the complete search strategy. Backward citations were also used to identify additional relevant articles resulting from the initial reference lists. The search was first performed in September 2021, and updated in June 2022. Studies published between 2007 and June 2022 were included in this review.

### 2.2 Study selection

The identified citations were imported into EndNote and de-duplicated (Bramer, 2016). Two reviewers read titles and abstracts (step one) and full-texts (step two) independently. AST or SP, and SLB performed the abstract screening, and subsequently, SLB and AST read the full-text studies. Disagreement among reviewers of the in−/exclusion of an abstract or full-test paper occurred was resolved by consensus discussions.

### 2.3 Inclusion criteria

Studies were included if the following criteria were met: (i) Using Personas defined as fictitious, specific, concrete representations of target users (Pruitt and Adlin, 2010) in the context of (ii) eHealth intervention, with eHealth referring to the use of information and communication technologies for health and health-related fields (WHO, 2022). eHealth intervention was defined as “treatment, typically behaviorally based, that is operationalized and transformed for delivery via the Internet” (p. 1) (Ritterband et al., 2006); ‘behavioral’ refers here to psychosocial interventions with elements of Cognitive Behavioral Therapy (CBT), Acceptance and Commitment Therapy (ACT), or other psychological approaches (e.g., psychoeducation, self-monitoring of psychosocial health aspects). Finally, the intervention had to target (iii) people with chronic pain defined as “pain that persists or recurs for longer than three months” (Treede et al., 2019), thus including different types of pain such as primary chronic pain, cancer, neuropathic, headache, or orofacial, visceral, and musculoskeletal pain.

Treatment approaches only focusing on physical aspects such as exercising/ physiotherapy or medication intake were excluded. Furthermore, telephone-, virtual reality-, video-only, CD-ROM interventions, and assistive technology (e.g., sensors) were excluded as they did not align with the eHealth intervention definition mentioned above. Studies had to be written in English and published in peer-reviewed journals. In previous systematic reviews focused on eHealth (Bartels et al., 2019; Christie et al., 2018), 2007 was set as a cut-off. In the present study, the 2007 cut off was also used because the first smartphone was released that year and the use of smart technologies significantly changed the use and possibilities of the internet.

### 2.4 Data extraction and synthesis

The PRISMA reporting structure guided data extraction (Page et al., 2021). Information on the study characteristics were extracted, i.e., target population, study aim, design, and intervention description. To answer the research question, data extraction focused specifically on the rationale for using Personas, how they were created and used, benefits and problems, and characteristics of Personas. A textual approach was chosen, specifically a narrative synthesis, to summarize information presented in the included studies and explain findings in a coherent way (Popay et al., 2019). As this study does not aim to determine the feasibility or effectiveness of the digital interventions itself, but instead focuses on methodology, neither a meta-analysis nor quality assessment of included studies was performed. This review is therefore classified as a scoping review (Munn et al., 2018; Peters et al., 2015).

## 3 Results

### 3.1 Review process

**Fig. 2:**
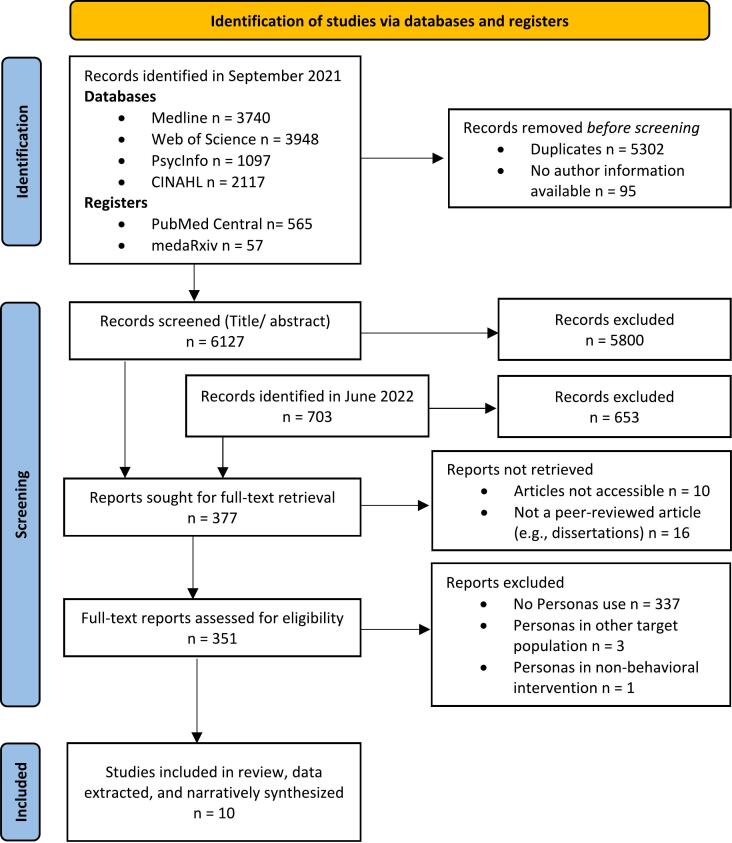
Flow-chart of review process from reference identification to inclusion for narrative synthesis.

In September 2021, 11,524 references were identified, leaving 6222 after de-duplication for the title and abstract screening. Additionally, 762 references were identified in June 2022 and a total of 6984 reports were screened. After excluding 6453 hits, 351 accessible and peer-reviewed reports were included in the full-text screening. Of these full-texts, 339 did not use Personas. Three articles used Personas in other target populations, i.e., prevention of pressure injuries in people with spinal cord injury (Amann et al., 2020), family-delivered eternal tube care (Cheng et al., 2020), and pediatric chronic illness care (Kaplan et al., 2017). One article used Personas in the context of non-behavioral eHealth interventions (Diet/ uric acid tracker in gout (Nguyen et al., 2018)). Ten articles met the inclusion criteria and were included in the present review (see Fig. 2).

### 3.2 General study characteristics

Descriptive information on the ten included studies is presented in Table 1. All studies were conducted in Europe and target pain populations include adults with chronic pain (primary musculoskeletal/ low back) (*n* = 6) (Bartels et al., 2022; Elbers et al., 2021; Gentili et al., 2020; Ledel Solem et al., 2020; Liu et al., 2020; Turesson et al., 2022), cancer pain (*n* = 2) (Hochstenbach et al., 2017; Rimmer et al., 2020), endometriosis (*n* = 1) (Schubert et al., 2022), or people at risk for chronic pain after total knee arthroplasty (n = 1) (Rognsvag et al., 2021). Studies described the planned (*n* = 3 protocol papers (Bartels et al., 2022; Rognsvag et al., 2021; Schubert et al., 2022)) or performed development of a behavioral eHealth intervention (based on e.g., CBT/ACT, digital self-monitoring of health aspects, goal-setting/ personal values, education, coping skills training).

**Table 1:** Study characteristics of pain-specific digital behavioral interventions using Personas.

#	Study	Target population	Study aim	Study design	Intervention
1	Bartels et al. (2022); Sweden	Chronic pain	(i) Develop, (ii) evaluate, and (iii) implement a digital behavioral treatment to improve well-being	Study protocol of a multi-phase project following the mHealth Agile Development and Evaluation Lifecycle	Internet-based platform (www.1177.se): Values-oriented exposure is the core process; treatment includes various aspects such as therapist contact, exercises (micro-sessions), validation/ appreciation, and extra material
2	Elbers et al. (2021); The Netherlands	Chronic musculoskeletal pain	(i) Provide an overview of activities during the course of a research project to develop a relapse prevention intervention for interdisciplinary pain treatment programs; (ii) Examine how co-design may contribute to stakeholder involvement, generation of relevant insights and ideas, and incorporation of stakeholder input into the intervention design	Single case study using the double diamond model	Solapp smartphone app: Provides patients with opportunities to describe important personal values and subsequently formulate related personal goals; app provides structure where patients can gradually progress towards each goal by means of planning specific ‘steps’
3	Gentili et al. (2020); Sweden	Chronic pain	(i) Document the development process (alpha and beta testing) and gained insights, to make this knowledge available to other developers and scientists; (ii) Evaluate and optimize the ACTsmart intervention (feasibility, usability)	Three development and evaluation phases (0–2) following the mHealth Agile Development and Evaluation Lifecycle	Smartphone-based ACTsmart treatment (based on ACT): Aims to improve functioning and quality of life by increasing psychological flexibility; micro-session format accessible through mobile device
4	Hochstenbach et al. (2017); The Netherlands	Outpatients with cancer pain	(i) Describe co-creation method directed towards the development of an eHealth intervention delivered by registered nurses to support self-management in outpatients with cancer pain	User-centered design (multidisciplinary team; iterative, incremental process)	Mobile application for patients and web application for nurses: morning diary (e.g., pain level, interference of pain); personalized day schedule and medication reminders; pain education and coping skills
5	Ledel Solem et al. (2020); Norway	Chronic pain	(i) Design and (ii) develop user-centered, evidence-based eHealth self-management intervention	Multidisciplinary and user-centered design approach	EPIO smartphone app: primarily CBT-based program, and aspects of ACT; educational topics, topic-related tasks, relaxation-focused exercises
6	Liu et al. (2020); France	Chronic low back pain	(i) Develop a mobile application dedicated to the management of chronic pain	User-centered approach for the design/ re-design of apps	MIA Healthcare app: Asks patients on a daily basis about characteristics/ factors related to their chronic pain (e.g., psychological state); monitor and advise patients; Chatbot; logbook for the health professional to better understand their patient's condition
7	Rimmer et al. (2020); the UK	Low- and intermediate-grade gliomas (also experiencing pain)	(i) Design an evidence-based and theoretically-informed supported self-management program to improve QoL in adults living with a specific form of brain tumor	Protocol of a multi-method study involving three sequential phases (in-depth interviews; co-design workshops; health economy assessment)	Self-management program (web-based or face-to-face): Content developed to support self-management, defined as an “individual's ability to manage the symptoms, treatment, physical and psychosocial consequences and lifestyle changes inherent in living with a chronic condition”. The self-management program will be the outcome of the trial and further tested
8	Rognsvag et al. (2021); Norway	(Patients at risk for) chronic pain following total knee arthroplasty	(i) Develop an internet-delivered iCBT program to combine exercise therapy and education	Two phases of the Medical Research Council framework for complex interventions (development, feasibility)	Internet-delivered iCBT program: Designed from relevant elements of the commercially-available Braive program
9	Schubert et al. (2022); Germany	Endometriosis	(i) Investigate the efficacy of internet-based CBT in improving health-related quality of life in patients with endometriosis	Study protocol of a monocentric randomized-controlled trial	Eight internet-based CBT modules: psychoeducation, cognitive restructuring, pacing, and emotion regulation; Weekly, written feedback from a trained therapist
10	Turesson et al. (2022); Sweden	Chronic pain (sustainable return-to-work process)	(i) Develop a digital support application with evidence-based content that includes a biopsychosocial perspective	User-centered agile design approach with a multidisciplinary project team	SWEPPE (Sustainable Worker Digital Support for Persons With Chronic Pain and Their Employers) app: Action plan including goal, challenges, and strategies; daily self-rating (e.g., pain, fatigue, sleep, depression, stress) and self-monitoring of progress; information on coping; employer's view with shared information

### 3.3 Using Personas when developing eHealth interventions for chronic pain

#### 3.3.1 Why were Personas used?

All included papers presented a rationale for why Personas were used in the project. Among the included papers, *general* reasons for using Personas when developing behavioral eHealth interventions for people with chronic pain included saving costs and resources during the design process (in terms of human, temporal, or financial (Liu et al., 2020)). Moreover, three papers stated that Personas can bring the user voice to the forefront and highlight areas of tension (Elbers et al., 2021; Ledel Solem et al., 2020; Liu et al., 2020). For the *development team*, the described rationales were to create empathy for and share an understanding of the user, facilitate discussions on important user characteristics and needs, and explore, guide, and validate ideas and solutions (Bartels et al., 2022; Elbers et al., 2021; Gentili et al., 2020; Hochstenbach et al., 2017; Ledel Solem et al., 2020; Liu et al., 2020; Rimmer et al., 2020; Turesson et al., 2022). Finally, Personas were seen as beneficial for *prospective users* (i.e., patients with chronic pain) as part of the intervention, as users could identify themselves with the Personas and Personas may highlight the relevance of the intervention or specific exercises (Rognsvag et al., 2021; Schubert et al., 2022). Details of the specific rationale for each study as well as information on the methodology to create Personas, characteristics of the Personas, and how they were used can be seen in Table 2.

**Table 2:** Personas use in the context of behavioral eHealth interventions for chronic pain as described in the included studies.

#	Study	Why were Personas used?	How were Personas created?	What do Pain Personas look like?	How were Personas used?
1	Bartels et al. (2022)	• Communicate user needs to the development team (Gentili et al., 2020; Silva and Teixeira, 2019) • Facilitate a more concrete discussion of patient needs and to what extent the treatment might match those needs (Miaskiewicz & Kozar, 2011) • Ensure that relevant characteristics and contextual factors are being considered (Børøsund et al., 2018)	Patient interviews in a previous study using Personas (Gentili et al., 2020) and discussed in online workshop; adjusted based on factors identified in research (Gerdle et al., 2019a; Gerdle et al., 2019b; Huijnen et al., 2015), other personas used in the region (no further details), and input from clinical researchers; edited over several month until the project partners agreed on a final version	Three personas (one presented in manuscript) with (i) personal information, including employment, education, family, background, social context, social support, and living area; (ii) patient pain profile, including pain problem, consequences, pain behavior, and attitude to treatment; (iii) healthcare and treatment, including contact with healthcare, comorbidities, and medicine, and (iv) personal needs and goals, specifically related to the treatment	Personas used in project identification phase; presented at the start of treatment workshops to discuss, for instance, if and how the treatment content and structure fits the personas’ characteristics and meet needs. Potential problems for Personas in relation to treatment elements were identified, resulting in further discussions and consensus-based adjustments
2	Elbers et al. (2021)	• Represent data as a coherent “whole” for usage throughout co-design activities • Highlight certain areas of tension • Facilitate discussions of important patient characteristics (Adlin et al., 2006)	Characteristics often discussed during previous patient interviews	Four Personas with variation on 2 characteristics: high vs low level of social support; high vs. low tendency to protect personal boundaries (Reflection of a distinct pattern in goals, attitudes, and behaviors based on empirical research among potential users)	Personas used in co-creation session: Students presented their final intervention concepts and stakeholders were asked to reflect on the concepts by taking patient perspectives into account
3	Gentili et al. (2020)	• Help designers create an understanding of the potential end user • Keep the end user in mind during the design process	Early user experience interviews with *n* = 9 individuals (age 19–65 years, 78 % women) with complex chronic pain	Three patient Personas describing demographic information, needs, and motivations, characteristics, and pain behaviors; one presented in manuscript; representation potential end-users	Personas used to build the interface (and continuously tested with the alpha users (interviewees)); no further details
4	Hochstenbach et al. (2017)	• Used to explore solutions	*–*	Representation of patient population (no further details); mentioned both Personas and scenarios	Personas used in phase 1, step 2 during brainstorm sessions (development team discussed research results in order to generate ideas and concepts)
5	Ledel Solem et al. (2020)	• Facilitate user engagement on basis of existing research recommendations (LeRouge et al., 2013) • Inform the development process and project team members about typical end users and their daily challenges, needs • Bring the patient's voices to the forefront in the development process	Previous findings in a qualitative study (Ledel Solem et al., 2019)	Five Personas and two patient journey maps (typical day in a patient's life); background information (i.e., stories to give each Persona depth), coping skills and everyday challenges, overview of technology skills, needs and requirements in relation to eHealth intervention	Personas used in workshops: Brief presentation of Personas/ journey maps at start of workshop; Personas were also updated based on participant feedback; 1st workshop: discussing Personas; 4th workshop: using Personas/ scenarios/ journey maps to discuss possibilities related to an eHealth self-management intervention and looking at potential barriers for use.
6	Liu et al. (2020)	• Share specific understanding and unique representation of each identified user group/ type of user • Guide/ validate/ prioritize suggested solutions and related IT developments, with regard to their adequacy for each Personas • Provide a human “face” in order to create empathy • Reduce costs (in terms of human, temporal and financial resources)	Quantitative (questionnaire *n* = 67) and qualitative (interview *n* = 12) studies conducted on the target population (patients/ physicians); clustering method to determine optimal number of and representative/ discriminatory variables for Personas	Two classes for patient Personas (real users): more intense and pervasive pain in class 1 than class 2, and more physician consultations in class 1 and class 2; Common values in both classes: pain duration >1 year, do sport min. Ones/ week, interested in sharing information about their pain	Personas used during (i) identification phase to understand the future users better and (ii) usability testing by creating scenarios, which were then rated by people with chronic pain (*n* = 10)
7	Rimmer et al. (2020)	• Generate intervention design ideas	(Not generated yet; no information on prospective process)	Representing different people affected by low- and intermediate-grade gliomas (Study protocol; Personas not developed yet)	Personas used during phase 2 in co-design workshops: Initial conceptualization of a supported self-management program. Workshop participants (approx. *n* = 15 survivors and informal caregivers) will be asked to consider what an intervention for each specific Persona might involve (e.g., components, mode of delivery)
8	Rognsvag et al. (2021)	• Represent a figure that osteoarthritis or total knee arthroplasty patients can identify with • Help patients see the relevance of the iCBT exercises	Based on typical osteoarthritis or total knee arthroplasty patient (no further details)	Representation of typical osteoarthritis or total knee arthroplasty patient: Persona undergoes either non-surgical treatment (Version A) or total knee arthroplasty surgery (Version B)	Personas used in most intervention modules (i.e., videos where patients follow the Persona)
9	Schubert et al. (2022)	• (In the intervention): Personas’ answers and thoughts on certain exercises are displayed as encouraging examples	–	Four Personas (no further details)	Personas used as part of intervention (i.e., to provide examples of responses from people with endometriosis to the iCBT exercises)
10	Turesson et al. (2022)	• Identify and fulfill the users’ needs and requirements	Previous research and clinical expertise with persons with chronic pain and other stakeholders (no further details)	One Persona and empathy map presented in manuscript (‘Carina’) for two different scenarios: (i) Being back at work; (ii) Using SWEPPE app	Personas/scenarios used during the product creation phase in workshops: two healthcare researchers and the software team verbally and visually presented information about different types of users and about how to bring the needs of the personas with chronic pain and their employers into the development process. For each scenario, brainstorming was performed. Outcomes: Two Empathy maps with statements of what the personas might think, feel, do, and say in a given situation; maps used to identify topics, questions, or needs to be considered while developing SWEPPE app

IT: information technology; iCBT: internet-based Cognitive behavioral therapy. SWEPPE: Sustainable Worker Digital Support for Persons With Chronic Pain and Their Employers.

#### 3.3.2 How were Personas created?

The process of creating Personas varied across studies. Two studies did not specify how Personas were created (Hochstenbach et al., 2017; Schubert et al., 2022) and two studies provided minimal detail, mentioning “typical patients” (Rognsvag et al., 2021), and “previous research and clinical expertise with persons with chronic pain and other stakeholders” (Turesson et al., 2022) as their source. Five studies used qualitative data from interviews to inform the creation of Personas (Bartels et al., 2022; Elbers et al., 2021; Gentili et al., 2020; Ledel Solem et al., 2020; Ledel Solem et al., 2019; Liu et al., 2020). In addition to qualitative methods, Bartels et al. (2022) used factors identified in research and input from clinical researchers, while Liu et al. (2020) included qualitative data from pain management questionnaires collected from both patients and healthcare professionals. The study of Rimmer et al. (2020) is ongoing and will generate Personas, but no information on the methodology was presented in the study protocol.

#### 3.3.3 What do Pain Personas look like?

Pain Personas characteristics and detailed descriptions were available in six of the ten studies included in this review (See Table 3). In one project, authors described planing to create Personas, but no characteristics were mentioned in the study protocol paper (Rimmer et al., 2020). Three studies did not provide details other than “four Personas are introduced in the beginning [of the intervention]; their answers and thoughts on certain exercises are displayed as encouraging examples”(Schubert et al., 2022), “represent the patient population” (Hochstenbach et al., 2017), and “based on typical osteoarthritis or total knee arthroplasty patient” (Rognsvag et al., 2021).

**Table 3:** Pain Personas: Summary of information included in the studies to describe the user.

Study	Sociodemographic background information	Example of thoughts^a^	Pain profile/behaviors	Needs and motivations	Technology use
Bartels et al. (2022)	✓		✓	✓	(✓)^b^
Elbers et al. (2021)		✓	✓	✓	
Gentili et al. (2020)	✓	✓	✓	✓	
Ledel Solem et al. (2020)	✓	✓	✓	✓	✓
Liu et al. (2020)	✓	✓	✓	✓	✓
Turesson et al. (2022)^c^	✓	✓	✓	✓	

a Referring to quotes or thought bubbles.

b Technology use only mentioned in one Persona not published in the manuscript.

c This study also included ‘Employer’ Personas.

Turesson et al. (2022) provided descriptions of scenarios and identified topics, questions, and needs for one Persona (‘Carina’). These scenarios included two situations (Being back at home vs. using the app-based intervention) and statements such as ‘Will I manage?’ or ‘How will SWEEPE help me return to work?’ (Turesson et al., 2022). Upon request, the authors provided their full Personas: a total of five Personas were used (three Personas with chronic pain and two employers). Pain Personas included a picture, name, job title, marriage status, and a narrative text about, for instance, origin of the pain, care, level of functioning, thoughts and worries around the pain and behavior. ‘Employer’ Personas also consisted of a picture, name, age, marital status, as well as a narrative on how they experience the situation and how they would like to support their employee with pain in the workplace.

Five studies included pictures of the Personas in their articles (Appendix B). Gentili et al. (2020) developed three Personas, with one being presented in the publication. This Persona (‘Stina’) included two thought bubbles, an icon of a person, as well as demographic information (e.g., age, living situation, marriage status), needs and motivations (e.g., needs a lot of feedback), characteristics (e.g., performance oriented), and pain behaviors (e.g., hard to sit still). Similarly, Bartels et al. (2022), building their three Personas on Gentili et al. (2020), presented one of three developed Personas (‘Aida’) in the publication. Characteristics reflected general information (e.g., age, employment, education, family, background and social context, social support, city/ country side), patient pain profile (e.g., pain problem, consequences, pain behavior, attitude to treatment), healthcare and treatment (e.g., contact with healthcare, comorbidities, medicine), and personal needs and goals (e.g., treatment needs and goals in relation to pain). One of their three Personas (64-year-old ‘Göran’, not published in the paper) includes information on technology use (i.e., unfamiliar, finds technology complicated and challenging).

Liu et al. (2020) presented ‘Sarah Roux’ in their manuscript, a Persona that was presented with a quote, a photo of a woman, sociodemographic information (e.g., age, employment status), characteristics (e.g., practices sports), motivation (e.g., often uses mobile apps), and pain management (e.g., takes pain medication frequently). This Persona represented the first group of patients (72.7 % of participants that completed questionnaires and interviews), who tend to have more intense and pervasive pain, consulted physicians more often, and were more interested in sharing information about their pain than the second group (27.3 % of participants). The second group was not presented as a figure, but was described as a group of patients that experienced lower levels of pain, contacted specialists less often, and engaged in sports slightly more often than the first group (Liu et al., 2020).

Ledel Solem et al. (2020) developed five Personas and two patient journey maps with one of each being presented in the manuscript. Personas included background information (i.e., stories to give more depth), coping skills, everyday challenges, overview of technological skills, needs, and requirements in relation to the eHealth intervention, while the journey maps demonstrated a ‘typical day’ in the person's life (Ledel Solem et al., 2020). Finally, Elbers et al. (2021) provided details on the Personas in the appendix: the four Personas were based on two characteristics: extrovert vs. introvert, and underuse vs. overuse, thus resulting in “the Social”, “the Dependent”, “the Perfectionist”, and “the Inactive”. The “pain path” of the Social Persona and a template for how the developed Solapp might be beneficial or hindering for these Personas were also presented (Elbers et al., 2021).

#### 3.3.4 How were Personas used?

Information on how Personas were used in the development of eHealth interventions was limited, with few details provided in all studies. Two studies described the Personas use briefly as “the interface was built based on Personas” (Gentili et al., 2020) or Personas were used for the “exploration of context” (Hochstenbach et al., 2017). Personas also helped to reflect on concepts by taking the patient perspective (Elbers et al., 2021), discuss potential possibilities or barriers of the intervention (Ledel Solem et al., 2020), or consider what the intervention might involve for the specific Personas (Rimmer et al., 2020). However, no details were mentioned to indicate in what setting these Personas-based discussions took place, who joined these conversations, if notes were taken, or if a guide was followed.

One study used Personas to better understand the future user and their interest in the app during the design process and also developed scenarios that were then rated by *n* = 10 patients in the usability testing (Liu et al., 2020). Moreover, Turesson et al. (2022) specified that the workshop included two health care researchers and the software team who brainstormed ideas with the outcome of empathy maps. These maps represent statements of what Personas might think, feel, do, and say in a given situation, and were used to identify topics, questions, or needs to be considered while developing SWEPPE. Furthermore, it was stated that Personas were presented at the start of workshops to discuss how treatment content and structure may fit patients, and to identify problems warranting potential adjustments (Bartels et al., 2022).

Finally, two studies included Personas in the intervention (but not explicitly in the development process): “Persona […] appears in all modules throughout the program” (Rognsvag et al., 2021) and “Personas are introduced at the beginning; their answers and thoughts on exercises are displayed as encouraging examples” (Schubert et al., 2022)). However, no details were presented.

## 4 Discussion

This article describes how Personas are used in the development of behavioral eHealth interventions for people with chronic pain. The systematic search resulted in the identification of ten peer-reviewed articles referring to “Pain Personas”. Articles were published between 2017 and 2022, including three study protocols, which highlights the timeliness of this review. All studies provided reasons for using Personas, namely general benefits such as saving resources and costs, benefits for the development team (i.e., shared an understanding of the target population), or benefits for the prospective patients when Personas were included in the intervention itself. Only half (*n* = 5) of the studies reported details on how Personas were created or what data Personas were based on. Characteristics of Personas differed slightly, with six studies describing Personas' pain profiles or pain-related behavior, personal needs and motivation, and five studies including sociodemographic and background information. Two studies elaborated on, or mentioned, the Persona's use of technology (Ledel Solem et al., 2020; Liu et al., 2020).

In summary, Personas is an innovative UX design methodology increasingly used for scientific innovation by pain researchers. However, a number of concerns regarding the use and reporting of Personas arise as a result of this review, including the scientific rigor of the methodology and level to which researchers reflect on and report the Personas process transparently. Furthermore, it remains understudied how effective Persona are as an aid to treatment development, what qualifies a Pain Persona as a ‘good one’, and clear steps for how to translate what Personas demonstrate into adjustments in eHealth products. These concerns will require further attention in the future.

### 4.1 Creating and using Pain Personas following the Personas Lifecycle

The transparency around the creation and use of Personas varied across studies. Half of the included studies reported how Personas were created or used (Bartels et al., 2022; Elbers et al., 2021; Gentili et al., 2020; Ledel Solem et al., 2020; Liu et al., 2020), while the other half only mentioned the term ‘Personas’ without details, making it impossible to understand or replicate their approach. In articles where the process was more thoroughly described, Personas were mainly based on qualitative data, with a few quantitative sources. Moreover, only two studies mentioned technology use of their Personas (Ledel Solem et al., 2020; Liu et al., 2020). In the context of eHealth, internet use, attitudes towards technology, and eHealth literacy (e.g., in older people) (Choi and DiNitto, 2013) can impact if a person will benefit from a digital intervention, and if these users will be reached. Therefore, technology use and digital literacy are important characteristics of Pain Personas and should be considered in future eHealth trials.

Overall, none of the studies explicitly followed an available guideline such as the Personas Lifecyle (Adlin and Pruitt, 2010) to guide, report, or review their Personas efforts. To follow the phases described in the Personas Lifecycle (i.e., family planning, conception and gestation, birth and maturation, adulthood, lifetime achievement and retirement) may increase scientific rigor and enhance transparency. Next to using this Lifecycle (Pruitt and Adlin, 2010), we propose a short version for pain researchers to stimulate more structured efforts of Pain Personas (Table 4). Researchers are invited to use this guideline, critically reflect and report on its implications, and thus contribute to increased scientific rigor when using Personas in pain research.

**Table 4:** Phases, considerations, and practical suggestions for the “Pain Personas Lifecycle” in the context of eHealth interventions; inspired by Adlin and Pruitt (2010).

Pain Personas Phase	Considerations	Practical suggestions
1. Identification (*Family Planning): Prior to any efforts, the purpose of and available material/ data for Personas is discussed, identified, and decided on.	o Who will be involved in and support the Personas efforts? o What is the main purpose of using Personas? o Why are Personas needed? o What data is available for Personas? o How and to whom will Personas be introduced?	Use Personas if: o You have established that it is the right method for your project (to guide the team, or to include Personas in the intervention). o The team understands and supports Personas efforts. o Data to inform Pain Personas can come from previous studies or newly collected information relevant to the specific context. o A solid plan for the next steps is prepared, and it suits the team and practical needs of the project.
2. Preparation and creation (*Conception and gestation): Clarify assumptions, process information, and create Personas.	o How many Personas will be necessary to communicate the main information from the data? o Which characteristics need to be presented and are of relevance? o Do the Personas reflect diversity (or patient sub-groups)? o How are Personas prioritized and validated? o How is decided that Personas are ready to be introduced to the team?	o Prepare Personas conceptually by identifying relevant categories (e.g., pain history, pain behaviors), processing data and available information, and outline Personas with specific data points and potentially refined sub-categories. o Create Personas by prioritizing key information and their specific relevance (e.g., main project aim); enrich Persona by adding individualized data including story-telling elements to give Personas a personality and context. Consider diversity in Personas to reflect the target population heterogeneously. o Validate Personas by double-checking it against data and/or with stakeholders. o Keep track of information not used, which would potentially reflect additional sub-groups. o Number of Personas: Usually three to five Personas are created; number depends on project goal, data/ information, and team engagement.
3. Introduction and maturation (*Birth and maturation): Make a Personas. plan and introduce Personas to team.	Ensure that the team knows: o What is the Personas method, and why is it used in this project? o Who are our Personas? o How were the Personas created, and why? o How should Personas be used in the design/ development process? Consider: o How does the team feel about using Personas?	Prepare/ follow a communication strategy (based on Phase 1): “Meet the people living with pain” o Introduce Personas method, reasons for creating them, and basic information how they were created and should be used to the core team. Allow for questions/ feedback/ reflections, to ensure that everyone is comfortable with the method. o Assign someone to maintain focus and help the team to include Personas in the design/ development process. o Different team members (e.g., designer, manager, IT) might need different information from Personas at different stages of the process (e.g., intervention concept, writing, testing).
4. Use (*Adulthood): Use Personas in specific ways in the design, development, evaluation, and release of the eHealth intervention.	Planning: o How do the pain Personas behave, think, or feel? What triggers, motivates, disturbs them? o Which Persona does the intervention (not) serve? Why/ why not? Exploring/ evaluating: o How would Personas experience the intervention? o Based on Personas, who will be the actual users during usability/ feasibility/ effectiveness trials? Release: o How can Personas support instructional material, guides, and editorial content?	o Remember: Personas are not perfect. o Bring Personas to meetings/ workshops. o Use prepared templates/ note taking to keep track of the information gained through Personas. o Summarize meetings and findings to generate key insights and brainstorm potential solutions. o Use Persona-weighted feature matrix and feature-value vs. technical feasibility plot (See Adlin and Pruitt, 2010) to determine which solutions should be prioritized and are feasible. o Create scenarios to understand Persona-intervention interaction. o Perform walk-throughs of Personas engaging with the intervention to determine potential benefits and issues. o Consider using Personas to inform recruitment of users, and reach of the intervention (e.g., how would Personas get in contact with the intervention?).
5. Evaluation and follow-up (*Lifetime achievement and retirement): Measure the success of Personas efforts and decide on reuse/ retirement of Personas.	o Were Personas perceived as helpful? o Has the team's focus on the users improved; if so, how (much)? o Has the development process improved; if so, how (much)? o Has the intervention improved; if so how (much)? o How much did the Personas effort cost? Were Personas worth the effort?	o Personas return of investment (ROI) can be measured (Souza et al., 2001). o Calculate time and money related to creating/ using Personas; consider potential delays of the project because of Personas. o List improvements made based on Personas, including intervention-related decisions, and impact on the final design. o Evaluate if Personas matched the actual users involved in the feasibility/ effectiveness trials. o Critically review and report Personas process transparently (e.g., in scientific publications). o If Personas should be reused: check if data is still valid and consider adjusting processes based on your lessons-learned.

Note: * refers to the original terminology proposed by Adlin and Pruitt (2010). This table does not aim to be exhaustive.

### 4.2 Promises and pitfalls of using Personas in pain research

In the included studies, researchers argue that Personas can be helpful for the development team, save costs, and be used in the intervention for patients to identify themselves with. These promises are in line with reasons mentioned in other fields, as described in the introduction. Notably, none of the included studies evaluated if their Pain Personas were actually effective and useful, and little is known about challenges and issues with creating and using Personas in pain research. However, one example of how effects of using Personas can be described is presented in Wärnestål et al. (2017), where effects of Personas as communication aid, idea generators, and Personas-driven design decisions are discussed.

Furthermore, it is noticeable that current Pain Personas show little diversity as most Personas were Caucasian (Appendix B) and only one mentioned a migration background (Bartels et al., 2022). While Personas aim to represent the “main target population”, this idea in itself appears exclusive to sub-groups and may lead to reduced representativeness and inclusivity. The chronic pain population is heterogeneous, and this heterogeneity should be reflected in Personas to tailor eHealth solutions to various needs. Diverse Personas can be seen, for instance, in Benedict et al. (2021), where six Personas depicting different “user types” are used to develop a decision aid and planning tool website for in vitro fertilization. More diversity is yet to be displayed in Pain Personas.

When looking at practical issues reported in other fields, it appears relevant that Personas are introduced from the beginning, and if team members are not familiar with this methodology, they may have little trust in its benefits (Blomquist and Arvola, 2002). It is therefore recommend that the use of Personas is advocated and all team members should be receptive of the method (Blomquist and Arvola, 2002). In the field of pain, little is known about pitfalls with Persons, and scientists are urged to critically review and report on Personas use by following available guidelines in the future, as described above.

### 4.3 No conscious patient representation, fictional patients, or patient involvement?

This article did not aim to compare different methodologies to develop eHealth interventions for people with chronic pain, and the use of Personas is one approach of many to creating a new smartphone app or website. Pain researchers also describe alternative development strategies, such as digitalizing a paper-based self-help book or treatment manual (Bendelin et al., 2018; Ljótsson et al., 2014), adapting or testing a digital treatment originally targeting another condition such as depression or anxiety (Blaney et al., 2021; Dear et al., 2018), relying on clinical expertise and knowledge of the research team (Bostrøm et al., 2020; Carpenter et al., 2012), and/or following theoretical frameworks (Darnall et al., 2020; Voerman et al., 2015). While all of these approaches individually and in combination may have their value, the needs of people with chronic pain might not be consciously operationalized, discussed, and considered in these processes. Instead, researchers may inadvertently use their own “internal Personas”; knowledge about or a memory of (experiences with) the target population. This intuitive approach may be cognitively biased (O'Sullivan and Schofield, 2018) or can comprise feasibility, usability, effectiveness, or implementation of eHealth at a later stage, which may be prevented with user voices actively represented.

As highlighted in the introduction, patient involvement when developing eHealth interventions is recommended. Five of the identified studies involved patients to create Personas, either by recruiting and interviewing patients directly (Gentili et al., 2020; Liu et al., 2020) or relying on previous qualitative information (Bartels et al., 2022; Elbers et al., 2021; Ledel Solem et al., 2020; Ledel Solem et al., 2019). This finding shows that Personas are not necessarily an alternative to patient involvement, but rather an additional means to represent users in decision-making processes and discussions throughout the development. People with chronic pain can certainly be engaged in a continuous co-creation or co-design processes (Cooke et al., 2021; Grasaas et al., 2019), or even potentially function as co-researchers to guide research questions and interpretation of results. Nevertheless, Personas simplify taking the patient perspective into account, when actual user involvement is challenging for practical, ethical, financial, or organizational reasons.

### 4.4 Strength and limitations of this review

This review provides a comprehensive and timely overview of the use of Personas in the context of eHealth interventions for chronic pain. A systematic search and double-reviewing process ensured that relevant literature was identified. In the 351 full-texts, alternative terminology (e.g., vignettes) was also considered (Castarlenas et al., 2015). This article can be the start of a Personas library and serve as an inspiration for pain researchers.

One limitation could be seen in the lack of quality assessment of the included studies in this review. However, as the focus of this review was on the Personas method rather than the quality of the trial itself, the approach taken does not limit the validity of the results. It is also possible that Personas were used in other eHealth interventions for chronic pain with a non-behavioral approach, but reviewing these manuscripts was beyond the scope of the present study. Furthermore, the suggested phases, considerations, and practical suggestions for Personas (Table 4) represent a preliminary guide based on the combination of the literature, on own experiences with Personas and the Personas Lifecycle (Adlin and Pruitt, 2010), and should thus be validated.

Moreover, Personas were here primarily viewed as a tool for designing and developing eHealth interventions. Similar design methodologies, such as empathy mapping (Siricharoen, 2021), were not identified through our systematic review but may also inform future projects. It is possible that authors used Personas without mentioning them in the manuscript, or using different terms, which may have prevented identification of relevant papers in this search. Reviewing the use of ‘avatars’ (i.e., figures or icons representing pain patients or therapists) or methodologies such as ‘patient narratives’ or ‘case stories’ might be of interest in the future as conceptual similarities to Personas appear. In one relatively recent study on the development of digital behavioral intervention for chronic pain, case stories were used as part of the treatment (Dear et al., 2015). While little information on the creation process or how these case stories were used is provided (i.e., ‘*case stories are provided, which describe how people with chronic pain apply the information and skills covered in the course*’ p.1925 (Dear et al., 2015)), the rationale overlaps with some of the identified Personas. A recent review argues furthermore that patient narratives can have several benefits for patients and healthcare professionals in providing health-related information (Drewniak et al., 2020). Thus, the purpose of patient narratives (e.g., inform, engage, model behavior) (Shaffer and Zikmund-Fisher, 2013) may overlap considerably with the purpose for using Personas. Although similar in several regards, differences appear. Personas are primarily a design tool, based on different sources (e.g., qualitative/ quantitative data) representing a group of users that share common features, and thus, are used by the development team to create a product or service, that matches the user's needs. Narratives, as well as case stories, might primarily be informed by one source (i.e., experience from one patient) to provide a story with a plot (Shen et al., 2015), commonly as part of a treatment or health communication tool, to broaden a patient's point of view and stimulate a behavioral change (Blenkiron, 2005). However, Personas can also be part of an intervention, as the present review shows, and it is generally possible to use case stories and narratives in the development phase of interventions. Therefore, it is likely that the terms are sometimes used interchangeably, and future studies should explore the exact overlap, methodological differences, and scientific implications.

## 5 Conclusions

Fictional patient characters, known as Personas, have been used as a methodology in ten recent studies developing eHealth interventions for chronic pain. While Personas appear innovative and generally considered to be beneficial, available guidelines are not followed and researchers do not critically review or report on the process of creating or using Personas. As “Pain Personas” are still a novel feature in scientific eHealth innovations, it is recommended for pain researchers to contribute to transparency by systematically assessing and reporting benefits and challenges with Personas, as well as making use of and evaluate design guidelines to enhance scientific rigor.

## Declaration of competing interest

The authors declare that they have no known competing financial interests or personal relationships that could have appeared to influence the work reported in this paper.

## Appendix A Supplementary data

Appendix A Documentation of search strategy. Appendix A

Appendix B Personas included in the identified studies, published in peer-reviewed journals. Appendix B
