# Supplementary material for: Using Personas in the development of eHealth interventions for chronic pain: A scoping review and narrative synthesis
Source: Internet Interv. 2023 Apr 3;32:100619. doi: 10.1016/j.invent.2023.100619 (PMC10235431; doi:10.1016/j.invent.2023.100619)
Supplement: Appendix A — Documentation of search strategy. [file mmc1.docx]

Documentation of search strategies

University Library search consultation group

Date: September 2021

Topic/research question: How are "personas" used in the development of eHealth interventions for chronic pain?

Name of researcher(s): Sara Bartels, Department of Clinical Neuroscience

Librarian(s): Emma-Lotta Säätelä

Databases:

1. Medline (Ovid)
2. Web of Science Core Collection (Clarivate)
3. PsycInfo (Ovid)
4. CINAHL (Ebsco)

Additional searches:

1. PubMed Central (ncbi.nlm.nih.gov/pmc)
2. medarXiv (medrxiv.org)

Total number of hits:

- Before deduplication: 11,524
- After deduplication: 6,222

Comments:

We use the method described by Bramer et al (2016) to remove duplicates in EndNote:

Bramer WM, Giustini D, de Jonge GB, Holland L, Bekhuis T. De-duplication of database search results for systematic reviews in EndNote. J Med Libr Assoc. 2016 Jul;104(3):240-3. doi: 10.3163/1536-5050.104.3.014.

1. Medline

| Interface: Ovid MEDLINE(R) and Epub Ahead of Print, In-Process & Other Non-Indexed Citations and Daily  Date of Search: 20 September 2021  Number of hits: 3,740  Comment: In Ovid, two or more words are automatically searched as phrases; i.e. no quotation marks are needed | Field labels   - exp/ = exploded MeSH term - / = non exploded MeSH term - .ti,ab,kf. = title, abstract and author keywords - adjx = within x words, regardless of order - * = truncation of word for alternate endings |
| --- | --- |
| Database(s): **Ovid MEDLINE(R) and Epub Ahead of Print, In-Process, In-Data-Review & Other Non-Indexed Citations and Daily**1946 to September 17, 2021 Search Strategy:   \| **#** \| **Searches** \| **Results** \| \| --- \| --- \| --- \| \| 1 \| Internet/ \| 76977 \| \| 2 \| Telemedicine/ \| 30114 \| \| 3 \| Mobile Applications/ \| 8589 \| \| 4 \| exp Computers, handheld/ \| 10275 \| \| 5 \| (app or apps or cellphone* or computer* or digital or internet* or ipad* or mobile application* or mobile device* or online or phone* or smartphone* or tablet* or technolog* or telephone* or video* or virtual or web based or web site or website).ti,ab,kf. \| 1489833 \| \| 6 \| (e health* or ehealth* or m health* or mhealth* or tele health* or telehealth* or tele medicine or telemedicine).ti,ab,kf. \| 36407 \| \| 7 \| or/1-6 \| 1533779 \| \| 8 \| exp Therapeutics/ \| 4828345 \| \| 9 \| Preventive Health Services/ \| 14075 \| \| 10 \| exp Primary prevention/ \| 162439 \| \| 11 \| Quaternary Prevention/ \| 4 \| \| 12 \| Secondary Prevention/ \| 21625 \| \| 13 \| Tertiary Prevention/ \| 182 \| \| 14 \| exp Health education/ \| 253602 \| \| 15 \| (intervention* or therap* or treat* or rehabilitat* or program* or prevent* or health promotion or health education or support or advice or consultation or counse?ling or training or coaching or motivational interview*).ti,ab,kf. \| 10408178 \| \| 16 \| or/8-15 \| 12704810 \| \| 17 \| 7 and 16 \| 779162 \| \| 18 \| Internet-Based Intervention/ \| 684 \| \| 19 \| Therapy, Computer-Assisted/ \| 6924 \| \| 20 \| Telerehabilitation/ \| 637 \| \| 21 \| exp Remote consultation/ \| 5378 \| \| 22 \| (distance counsel?ing or distance consultation* or e consultation* or econsultation* or e counsel?ing or ecounsel?ing or e therapies or e therapy or etherap* or e visit* or evisit* or mobile counsel?ing or mobile consultation* or remote consultation* or remote counsel?ing or tele consultation* or teleconsultation* or tele rehabilitation or telerehabilitation).ti,ab,kf. \| 4366 \| \| 23 \| or/18-22 \| 16407 \| \| 24 \| 17 or 23 \| 785106 \| \| 25 \| Chronic pain/ \| 17711 \| \| 26 \| ((chronic or long standing or long term or widespread or wide spread or recurring or reoccurring) adj3 pain).ti,ab,kf. \| 75642 \| \| 27 \| 25 or 26 \| 78635 \| \| 28 \| 24 and 27 \| 4554 \| \| 29 \| limit 28 to english language \| 4365 \| \| 30 \| limit 29 to yr="2007-Current" \| 3740 \| | |

2. Web of Science Core Collection

| Interface: Clarivate Analytics  Editions: A&HCI, ESCI, SCI-EXPANDED, SSCI  Date of Search: 20 September 2021  Number of hits: 3,948 | Field labels   - TS/Topic = title, abstract, author keywords and Keywords Plus - NEAR/x = within x words, regardless of order - * = truncation of word for alternate endings - Exact search= no lemmatization |
| --- | --- |
| **History**  11  **#7 AND #8** and **2007** or **2008** or **2009** or **2010** or **2011** or **2012** or **2013** or **2014** or **2015** or **2016** or **2017** or **2018** or **2019** or **2020** or **2021** (Publication Years) and **English** (Languages)  Edit  \| Exact search  Add to Search  [3,948](https://www.webofscience.com/wos/woscc/summary/a0009621-8bf1-4b9f-8aa7-9a9ce1222135-091f6af1/relevance/1)  10  **#7 AND #8** and **2007** or **2008** or **2009** or **2010** or **2011** or **2012** or **2013** or **2014** or **2015** or **2016** or **2017** or **2018** or **2019** or **2020** or **2021** (Publication Years)  Edit  \| Exact search  Add to Search  [4,057](https://www.webofscience.com/wos/woscc/summary/067be86e-8f43-4348-a55e-d7e1c160ba9f-091f681f/relevance/1)  9  **#7 AND #8**  Edit  \| Exact search  Add to Search  [4,645](https://www.webofscience.com/wos/woscc/summary/8e09fa6c-e179-494c-a9e4-aaf8f4821252-091f5fa7/relevance/1)  8  **TS=((chronic or "long standing" or "long term" or widespread or "wide spread" or recurring or reoccurring) NEAR/2 pain)**  Edit  \| Exact search  Add to Search  [83,199](https://www.webofscience.com/wos/woscc/summary/d1eb9499-6e1c-479c-827e-af6266168edb-091f5e22/relevance/1)  7  **#5 OR #6**  Edit  \| Exact search  Add to Search  [1,107,706](https://www.webofscience.com/wos/woscc/summary/47218240-ad8d-4afb-914f-d8e040356eb5-091f5ca4/relevance/1)  6  **TS=("distance counsel$ing" or "distance consultation*" or "e consultation*" or econsultation* or "e counsel$ing" or ecounsel$ing or "e therapies" or "e therapy" or etherap* or "e visit*" or evisit* or "mobile counsel$ing" or "mobile consultation*" or "remote consultation*" or "remote counsel$ing" or "tele consultation*" or teleconsultation* or "tele rehabilitation" or telerehabilitation)**  Edit  \| Exact search  Add to Search  [5,154](https://www.webofscience.com/wos/woscc/summary/6993ae26-e04b-4469-8098-992c9bc73c03-091f5b3d/relevance/1)  5  **#3 AND #4**  Edit  \| Exact search  Add to Search  [1,105,766](https://www.webofscience.com/wos/woscc/summary/7b51ddbe-caa1-49a5-a471-f66d83120c26-091f58a1/relevance/1)  4  **TS=(intervention* or therap* or treat* or rehabilitat* or program* or prevent* or "health promotion" or "health education" or support or advice or consultation or counse$ling or training or coaching or "motivational interview*")**  Edit  \| Exact search  Add to Search  [12,816,930](https://www.webofscience.com/wos/woscc/summary/c252dcdb-8c49-42f8-991c-8347b83c920f-091f55b4/relevance/1)  3  **#1 OR #2**  Edit  \| Exact search  Add to Search  [3,551,125](https://www.webofscience.com/wos/woscc/summary/b886f99e-3ccc-4d0f-9091-1f2146dd463b-091f5452/relevance/1)  2  **TS=("e health*" or ehealth* or "m health*" or mhealth* or "tele health*" or telehealth* or "tele medicine" or telemedicine)**  Edit  \| Exact search  Add to Search  [43,082](https://www.webofscience.com/wos/woscc/summary/87b79f8a-311d-4913-b271-145194e1aeb0-091f4914/relevance/1)  1  **TS=(app or apps or cellphone* or computer* or digital or internet* or ipad* or "mobile application*" or "mobile device*" or online or phone* or smartphone* or tablet* or technolog* or telephone* or video* or virtual or "web based" or "web site" or website)**  Edit  \| Exact search  Add to Search  [3,535,319](https://www.webofscience.com/wos/woscc/summary/d74a0cd4-bf57-4288-9c24-022639c3cd51-091f471d/relevance/1) | |

3. Psycinfo

| Interface: Ovid  Date of Search: 20 September 2021  Number of hits: 1,097  Comment: In Ovid, two or more words are automatically searched as phrases; i.e. no quotation marks are needed | Field labels   - exp/ = exploded controlled term - / = non exploded controlled term - .ti,ab,id. = title, abstract and author keywords - adjx = within x words, regardless of order - * = truncation of word for alternate endings |
| --- | --- |
| Database(s): **APA PsycInfo**1806 to September Week 2 2021 Search Strategy:   \| **#** \| **Searches** \| **Results** \| \| --- \| --- \| --- \| \| 1 \| exp internet/ \| 30337 \| \| 2 \| mobile applications/ \| 1351 \| \| 3 \| exp mobile devices/ \| 9125 \| \| 4 \| (app or apps or cellphone* or computer* or digital or internet* or ipad* or mobile application* or mobile device* or online or phone* or smartphone* or tablet* or technolog* or telephone* or video* or virtual or web based or web site or website).ti,ab,id. \| 447600 \| \| 5 \| (e health* or ehealth* or m health* or mhealth* or tele health* or telehealth* or tele medicine or telemedicine).ti,ab,id. \| 7419 \| \| 6 \| or/1-5 \| 451536 \| \| 7 \| exp prevention/ \| 65567 \| \| 8 \| exp health education/ \| 19577 \| \| 9 \| exp treatment/ \| 1109074 \| \| 10 \| (intervention* or therap* or treat* or rehabilitat* or program* or prevent* or health promotion or health education or support or advice or consultation or counse?ling or training or coaching or motivational interview*).ti,ab,id. \| 2105329 \| \| 11 \| or/7-10 \| 2361224 \| \| 12 \| 6 and 11 \| 229709 \| \| 13 \| exp computer assisted therapy/ \| 11027 \| \| 14 \| (distance counsel?ing or distance consultation* or e consultation* or econsultation* or e counsel?ing or ecounsel?ing or e therapies or e therapy or etherap* or e visit* or evisit* or mobile counsel?ing or mobile consultation* or remote consultation* or remote counsel?ing or tele consultation* or teleconsultation* or tele rehabilitation or telerehabilitation).ti,ab,id. \| 864 \| \| 15 \| 13 or 14 \| 11305 \| \| 16 \| 12 or 15 \| 230403 \| \| 17 \| chronic pain/ \| 14466 \| \| 18 \| ((chronic or long standing or long term or widespread or wide spread or recurring or reoccurring) adj3 pain).ti,ab,id. \| 22748 \| \| 19 \| or/17-18 \| 24020 \| \| 20 \| 16 and 19 \| 1376 \| \| 21 \| limit 20 to yr="2007 -Current" \| 1160 \| \| 22 \| limit 21 to english language \| 1097 \| | |

4. Cinahl

| Interface: Ebsco  Date of Search: 20 September 2021  Number of hits: 2,117 | Field labels   - MH+ = exploded Cinahl Heading - MH = non exploded Cinahl Heading - TI = title - AB = abstract - Nx = within x words, regardless of order - * = truncation of word for alternate endings |
| --- | --- |
| \| **#** \| **Query** \| **Results** \| \| --- \| --- \| --- \| \| S25 \| S19 AND S22  Limiters - Published Date: 20070101-20211231 Narrow by Language: - english \| 2,117 \| \| S24 \| S19 AND S22  Limiters - Published Date: 20070101-20211231 \| 2,148 \| \| S23 \| S19 AND S22 \| 2,456 \| \| S22 \| S20 OR S21 \| 46,110 \| \| S21 \| TI ( ((chronic OR "long standing" OR "long term" OR widespread OR "wide spread" OR recurring or reoccurring ) N3 pain ) ) OR AB ( ((chronic OR "long standing" OR "long term" OR widespread OR "wide spread" OR recurring or reoccurring ) N3 pain ) ) \| 37,864 \| \| S20 \| (MH "Chronic Pain") \| 23,972 \| \| S19 \| S13 OR S18 \| 280,153 \| \| S18 \| S14 OR S15 OR S16 OR S17 \| 22,924 \| \| S17 \| TI ( "distance counsel#ing" OR "distance consultation*" OR "e consultation*" OR econsultation* OR "e counsel#ing" OR ecounsel#ing OR "e therapies" OR "e therapy" OR etherap* OR "e visit*" OR evisit* OR "mobile counsel#ing" OR "mobile consultation*" OR "remote consultation*" OR "remote counsel#ing" OR "tele consultation*" OR teleconsultation* OR "tele rehabilitation" OR telerehabilitation ) OR AB ( "distance counsel#ing" OR "distance consultation*" OR "e consultation*" OR econsultation* OR "e counsel#ing" OR ecounsel#ing OR "e therapies" OR "e therapy" OR etherap* OR "e visit*" OR evisit* OR "mobile counsel#ing" OR "mobile consultation*" OR "remote consultation*" OR "remote counsel#ing" OR "tele consultation*" OR teleconsultation* OR "tele rehabilitation" OR telerehabilitation ) \| 1,664 \| \| S16 \| (MH "Telemedicine+") \| 16,653 \| \| S15 \| (MH "Therapy, Computer Assisted") \| 5,425 \| \| S14 \| (MH "Internet-Based Intervention") \| 239 \| \| S13 \| S7 AND S12 \| 269,270 \| \| S12 \| S8 OR S9 OR S10 OR S11 \| 3,418,607 \| \| S11 \| TI ( intervention* or therap* or treat* or rehabilitat* or program* or prevent* or "health promotion" or "health education" or support or advice or consultation or counse#ling or training or coaching or "motivational interview*" ) OR AB ( intervention* or therap* or treat* or rehabilitat* or program* or prevent* or "health promotion" or "health education" or support or advice or consultation or counse#ling or training or coaching or "motivational interview*" ) \| 2,542,659 \| \| S10 \| (MH "Health Education") \| 28,001 \| \| S9 \| (MH "Preventive Health Care") \| 21,433 \| \| S8 \| (MH "Therapeutics+") \| 1,604,413 \| \| S7 \| S1 OR S2 OR S3 OR S4 OR S5 OR S6 \| 491,864 \| \| S6 \| TI ( "e health*" or ehealth* or "m health*" or mhealth* or "tele health*" or telehealth* or "tele medicine" or telemedicine ) OR AB ( "e health*" or ehealth* or "m health*" or mhealth* or "tele health*" or telehealth* or "tele medicine" or telemedicine ) \| 17,377 \| \| S5 \| TI ( app or apps or cellphone* or computer* or digital or internet* or ipad* or "mobile application*" or "mobile device*" or online or phone* or smartphone* or tablet* or technolog* or telephone* or video* or virtual or "web based" or "web site" or website ) OR AB ( app or apps or cellphone* or computer* or digital or internet* or ipad* or "mobile application*" or "mobile device*" or online or phone* or smartphone* or tablet* or technolog* or telephone* or video* or virtual or "web based" or "web site" or website ) \| 454,924 \| \| S4 \| (MH "Computers, Hand-Held+") \| 7,774 \| \| S3 \| (MH "Mobile Applications") \| 9,444 \| \| S2 \| (MH "Telehealth") OR (MH "Telenursing") OR (MH "Telepsychiatry") \| 13,418 \| \| S1 \| (MH "Internet") \| 51,885 \| | |

5. PubMed Central

| Interface: https://www.ncbi.nlm.nih.gov/pmc/  Date of Search: 20 September 2021  Number of hits: 565 | Field labels   - Body - All words = fulltext article - MeSH-terms=exploded MeSH-term |
| --- | --- |
| \| Search \| Query \| Items found \| \| --- \| --- \| --- \| \| #[6](https://www.ncbi.nlm.nih.gov/pmc/advanced) \| Search **(#3 AND #4)** Sort by: **PubDate** Filters: **Publication date from 2007/01/01 to 2021/12/31** \| [565](https://www.ncbi.nlm.nih.gov/pmc/?cmd=HistorySearch&querykey=6) \| \| #5 \| Search **(#3 AND #4)** \| [743](https://www.ncbi.nlm.nih.gov/pmc/?cmd=HistorySearch&querykey=5) \| \| [#4](https://www.ncbi.nlm.nih.gov/pmc/advanced) \| Search **(persona[Body - All Words] OR personas[Body - All Words])** \| [6474](https://www.ncbi.nlm.nih.gov/pmc/?cmd=HistorySearch&querykey=4) \| \| [#3](https://www.ncbi.nlm.nih.gov/pmc/advanced) \| Search **(#1 OR #2)** \| [693570](https://www.ncbi.nlm.nih.gov/pmc/?cmd=HistorySearch&querykey=3) \| \| [#2](https://www.ncbi.nlm.nih.gov/pmc/advanced) \| Search **chronic pain[MeSH Terms]** \| [4644](https://www.ncbi.nlm.nih.gov/pmc/?cmd=HistorySearch&querykey=2) \| \| [#1](https://www.ncbi.nlm.nih.gov/pmc/advanced) \| Search **((chronic OR "long term" OR "long standing" or "wide spread" OR widespread OR recurring OR reoccurring)) AND pain** \| [693570](https://www.ncbi.nlm.nih.gov/pmc/?cmd=HistorySearch&querykey=1) \| | |

6. medarXiv

| Interface: medarxiv.org  Date of Search: 20 September 2021  Number of hits: 57 |  |
| --- | --- |
| full text or abstract or title *persona personas* (match whole any) | |
