# Supplementary material for: Using Personas in the development of eHealth interventions for chronic pain: A scoping review and narrative synthesis
Source: Internet Interv. 2023 Apr 3;32:100619. doi: 10.1016/j.invent.2023.100619 (PMC10235431; doi:10.1016/j.invent.2023.100619)
Supplement: Appendix B — Personas included in the identified studies, published in peer-reviewed journals. [file mmc2.docx]

Appendix B. Personas included in the identified studies, published in peer-reviewed journals.

a.

b.

c.

d.

e.

Personas used in the development of eHealth interventions for chronic pain. a.: Gentili et al. (2020); b.: Bartels et al. (2022); c.: Liu et al. (2020); d.: Ledel Solem et al. (2020); e.: Elbers et al. (2021). The corresponding authors gave their written permission for these Figures to be included here.

**References**

- Gentili, C., Zetterqvist, V., Rickardsson, J., Holmström, L., Simons, L. E., & Wicksell, R. K. (2020). ACTsmart–development and feasibility of digital acceptance and commitment therapy for adults with chronic pain. NPJ digital medicine, 3(1), 1-12. <https://www.nature.com/articles/s41746-020-0228-4>
- Bartels, S. L., Johnsson, S. I., Boersma, K., Flink, I., McCracken, L. M., Petersson, S., Christie, H. L., Feldman, I., Simons, L. E., Onghena, P., Vlaeyen, J. W. S., & Wicksell, R. K. (2022). Development, evaluation and implementation of a digital behavioural health treatment for chronic pain: study protocol of the multiphase DAHLIA project. BMJ open, 12(4), e059152. <https://doi.org/10.1136/bmjopen-2021-059152>
- Liu, Y. D., Encelle, B., & Sehaba, K. (2020). A user-centered approach to design a mobile application for chronic pain management. Modelling, Measurement and Control C, 81(1-4), 24-29. <https://doi.org/https://doi.org/10.18280/mmc_c.811-405>
- Ledel Solem, I. K., Varsi, C., Eide, H., Kristjansdottir, O. B., Mirkovic, J., Børøsund, E., Haaland-Øverby, M., Heldal, K., Schreurs, K. M., Waxenberg, L. B., Weiss, K. E., Morrison, E. J., & Solberg Nes, L. (2019). Patients' Needs and Requirements for eHealth Pain Management Interventions: Qualitative Study. J Med Internet Res, 21(4), e13205. <https://doi.org/10.2196/13205>
- Elbers, S., van Gessel, C., Renes, R. J., van der Lugt, R., Wittink, H., & Hermsen, S. (2021). Innovation in pain rehabilitation using co-design methods during the development of a relapse prevention intervention: case study. Journal of medical Internet research, 23(1), e18462. <https://www.jmir.org/2021/1/e18462/>
